# Contemporary Theory and Research

**Published:** 1893-03-18

**Authors:** 


### CONTEMPORARY THEORY AND RESEARCH.

### ANTIDOTES TO PHOSPHORUS.

Dr. Thornton communicates to the *New York Therapeutic Gazette* the result of his experiments with respect to phosphorus poisoning. He says: "The antidotes recommended in many text-books are old French oil of turpentine or sulphate of copper. Antal, of Buda-Pesth, has recently experimented with, and highly recommended, permanganate of potassium. Solution of hydrogen peroxide has been suggested. Old French oil of turpentine, which is acid in reaction, is said to form with phosphorus a crystalline and spermaceti-like mass, which has been called 'turpentine phosphoric acid.' The ordinary oil of turpentine does not bring about this change, and inasmuch as the old French oil of turpentine cannot be obtained, it should cease to be considered as a practical antidote. When a solution of sulphate of copper is poured over phosphorus, the latter becomes at once coated with a black deposit of copper, while phosphoric and sulphuric acid are found in the solution. The same reaction occurs instantly upon mixing solutions of the two substances, one grain of phosphorus reducing about four and a half grains of sulphate of copper. In all cases of phosphorus poisoning in which sulphate of copper was used as an antidote, death resulted. I am of the opinion it should cease to be recommended. The mixing of solutions of phosphorus and hydrogen peroxide result in the formation of phosphoric acid in the solution. The animal to which the solution of hydrogen peroxide was administered recovered after phosphorus poisoning. Unchanged phosphorus was vomited and passed by the bowels by this animal, and severe gastro enteritis and exhaustion resulted. Peroxide of hydrogen is too slow in oxidising the phosphorus and too irritating upon the digestive tract to be a valuable antidote. Solutions of phosphorus and permanganate of potassium, when shaken together, precipitate a black oxide of manganese, phosphoric acid and phosphates being found in solution. A few drops of dilute hydrochloric acid hastens this change, chloride of manganese being formed. The conclusion arrived at is: Permanganate of potassium is the best antidote for phosphorus. It must be used before the poison has become absorbed, and must be well diluted (·5 to 1 per cent. solution), or vomiting will result before the chemical reaction has taken place in the stomach. It must be given in excess, as considerable permanganate is reduced by the organic substances in the stomach."

### TRIONAL, THE NEW HYPNOTIC.

As "trional" seems to be a hypnotic of real value, worthy of the practitioner's attention, we furnish in abridged form the conclusions arrived at with regard to it by M. Boettiger. He is convinced by considerable experience that "trional" is at once a powerful hypnotic and sedative, which, used in certain doses, provokes but very rarely any disagreeable symptoms, and then without any serious consequences (sensations of fatigue, faintness, or giddiness). As a soporific the drug acts very rapidly, often at the end of a quarter of an hour, so that trional may be administered at the moment of retiring to rest. In simple sleeplessness, a dose of 15 grains is often sufficient to induce sleep; a dose of 30 grains acts without fail. In cases of slight psychic excitement and emotional troubles, primitive or secondary, trional proves very efficacious as a hypnotic. Trional has only failed in M. Boettiger's hands in the case of patients suffering from violent neuralgic pain, extreme mental disturbance, or the effects of drink. The hypnotic dose of trional should never exceed three grammes (45 grains).

